# Different dimensions of wellness in drug addiction treatment

**DOI:** 10.1192/j.eurpsy.2022.2146

**Published:** 2022-09-01

**Authors:** M. Delic

**Affiliations:** University Psychiatric Clinic Ljubljana, Center For Treatment Of Drug Addiction, Ljubljana, Slovenia

**Keywords:** wellness, well-being, Treatment, drug addiction

## Abstract

**Introduction:**

According to the World Health Organisation goals of treatment of drug addiction are: reducing of drug use and craving, improving of health, well-being and social functioning of the affected individual, prevention of future harms by decreasing the risk of complications and relapse. Wellness means a sense of overall well-being incorporating numerous aspects of an individual’s life. These include physical, mental, emotional, intellectual, occupational, and spiritual aspects. For those who suffer from mental and substance use disorders, wellness means feeling a sense of purpose in life, being actively involved in work or play that is satisfying, finding happiness, having joyful relationships, and having a healthy body and living environment. .

**Objectives:**

We will present different dimensions of wellness and describe how to incorporate these dimensions into drug addiction treatment.

**Methods:**

Presentation of theoretical frame and description of treatment programme at the Center for Treatment of Drug Addiction Ljubljana.

**Results:**

When each of wellness dimensions is balanced, it is easier to maintain recovery process and avoid the triggers of relapse.

**Conclusions:**

In the context of wellness, treatment goal is to maximize the capacity of person to feel, think and act in ways that enhance his/her ability to enjoy life and deal with the challenges he/she face. Wellness lifestyle includes a balance of health habits such as adequate sleep and rest, productivity, exercise, participation in meaningful activity, nutrition, productivity, social contact, and supportive relationships.

**Disclosure:**

No significant relationships.

